# Functions of Ubiquitin and SUMO in DNA Replication and Replication Stress

**DOI:** 10.3389/fgene.2016.00087

**Published:** 2016-05-13

**Authors:** Néstor García-Rodríguez, Ronald P. Wong, Helle D. Ulrich

**Affiliations:** Institute of Molecular BiologyMainz, Germany

**Keywords:** ubiquitin, SUMO, DNA replication, DNA replication stress, DNA damage, DNA repair, genome stability

## Abstract

Complete and faithful duplication of its entire genetic material is one of the essential prerequisites for a proliferating cell to maintain genome stability. Yet, during replication DNA is particularly vulnerable to insults. On the one hand, lesions in replicating DNA frequently cause a stalling of the replication machinery, as most DNA polymerases cannot cope with defective templates. This situation is aggravated by the fact that strand separation in preparation for DNA synthesis prevents common repair mechanisms relying on strand complementarity, such as base and nucleotide excision repair, from working properly. On the other hand, the replication process itself subjects the DNA to a series of hazardous transformations, ranging from the exposure of single-stranded DNA to topological contortions and the generation of nicks and fragments, which all bear the risk of inducing genomic instability. Dealing with these problems requires rapid and flexible responses, for which posttranslational protein modifications that act independently of protein synthesis are particularly well suited. Hence, it is not surprising that members of the ubiquitin family, particularly ubiquitin itself and SUMO, feature prominently in controlling many of the defensive and restorative measures involved in the protection of DNA during replication. In this review we will discuss the contributions of ubiquitin and SUMO to genome maintenance specifically as they relate to DNA replication. We will consider cases where the modifiers act during regular, i.e., unperturbed stages of replication, such as initiation, fork progression, and termination, but also give an account of their functions in dealing with lesions, replication stalling and fork collapse.

## Introduction

DNA replication in eukaryotes is a multi-step process that is tightly coupled to both cell cycle progression and the DNA damage response (Leman and Noguchi, 2013; Siddiqui et al., 2013; Berti and Vindigni, 2016). After completion of mitosis during the G1 stage of the cell cycle, replication origins are prepared for activation in a process called origin licensing (Siddiqui et al., 2013). This reaction results in the formation of pre-replicative complexes (pre-RCs) at replication origins, which include key components of the replicative helicase, albeit in an inactive form. Licensing restricts origin firing to once per cell cycle, thus preventing genome instability induced by re-replication. At the entry into S phase, DNA replication is initiated by the action of cell cycle-regulated kinases, resulting in the activation of the replicative helicase and the separation of strands to form the first replication forks. This is helicase and several DNA polymerases, but also a large number of accessory factors responsible accompanied by the assembly of replisomes, multi-protein complexes that comprise not only the for monitoring replication fork progression, generating checkpoint and damage signals, and coordination of DNA synthesis with chromatin assembly (Leman and Noguchi, 2013). In eukaryotes, origin firing follows a temporally regulated program throughout S phase, giving rise to distinct early- and late-replicating regions of the genome (Renard-Guillet et al., 2014). The pattern of origin firing is flexible and reacts to situations such as the stalling of individual forks or the perception of a global damage signal by the cell. DNA synthesis proceeds bi-directionally, initiated by the deposition of short RNA primers that are subsequently extended by DNA polymerase α. Leading and lagging strand replication by the main replicative DNA polymerases 𝜀 and δ, respectively, is closely coordinated with the unwinding of the template DNA. As a consequence, accumulation of extended regions of single-stranded (ss)DNA is perceived as a sign of fork stalling and triggers a checkpoint response that suppresses the firing of late replication origins and prevents entry into mitosis (Leman and Noguchi, 2013). The nicks in the emerging lagging strand, arising from its discontinuous synthesis, are successively sealed by DNA ligase. As replication units (replicons) from neighboring origins meet, replication forks merge and replication is terminated by the disassembly of the replisomes. Since DNA replication takes place in the context of chromatin, removal of nucleosomes in front of the helicase and their renewed deposition after passage of the replication fork need to be synchronized with DNA synthesis (Groth, 2009). This coordination, actively mediated by components of the replisome, also protects against the loss of epigenetic marks during replication.

Accurate control over all stages of DNA replication is of vital importance for the maintenance of genome integrity in proliferating cells. Both incomplete replication and over-replication interfere with proper chromosome segregation, and defects in replication fidelity pose a serious threat to genome stability due to an increased mutation load. Hence, the mechanisms ensuring complete and accurate replication need to be considered as part of a cell’s repertoire to defend itself against insults to its genome. By reversibly altering the properties of their target proteins, various different posttranslational protein modifications contribute significantly to these processes. Over the past decade, we have witnessed the emergence of ubiquitin and SUMO as key regulators of genome maintenance pathways (Ulrich and Walden, 2010; Jackson and Durocher, 2013). Although best known for mediating protein degradation, ubiquitin can convey a variety of non-proteolytic signals. This can partly be ascribed to the effects of mono-ubiquitylation, but also to ubiquitin’s ability to form polymeric chains of different geometries, recognized by highly chain-selective effector proteins (Komander and Rape, 2012). More recently, it has been realized that SUMO can also trigger degradation of its targets by forming polymeric SUMO chains interacting with a class of enzymes known as SUMO-targeted ubiquitin ligases (STUbLs; Prudden et al., 2007; Sriramachandran and Dohmen, 2014). Hence, both proteolytic and non-proteolytic contributions need to be considered in discussing the effects of ubiquitin and SUMO on DNA replication.

This review will cover the functions of ubiquitin and SUMO during unperturbed replication, i.e., during origin licensing, replication elongation and termination, and with regard to chromatin assembly and nuclear structure. Another important aspect will be the response to replication stress. As much of the recent progress in the field can be ascribed to large-scale siRNA screens and proteomic approaches, mechanistic information is often lagging behind the identification of novel modification targets and conjugation factors. We will therefore refrain from giving a comprehensive account of all the enzymes and substrates involved in DNA replication and rather focus on representative examples where a relevant functional context is available.

## Replication of Intact DNA

The function of ubiquitylation in unperturbed DNA replication has been the subject of an excellent recent review (Moreno and Gambus, 2015), to which the reader is referred for details, particularly with respect to proteolytic functions of ubiquitylation. Here we complement this with information on the roles of protein SUMOylation, and we discuss the recurring problem of distinguishing modifications that are inherently part of the replication process from those occurring in response to spontaneous problems based on difficult-to-replicate sequences or chromatin regions.

### Contributions of Ubiquitin and SUMO to Origin Licensing and Replication Initiation

At the entry into S phase, ubiquitin functions predominantly as an inducer of proteasomal degradation, owing to its prominent role in cell cycle regulation (Teixeira and Reed, 2013). Preparation for DNA replication requires loading of the hexameric ring-shaped Mcm2-7 complex onto origins of replication, mediated by the origin recognition complex (ORC) and two auxiliary factors, Cdt1 and Cdc6 (Siddiqui et al., 2013). Establishment of the pre-RC can only proceed late in mitosis and during G1 phase, when cyclin-dependent kinase (CDK) levels are low. This is achieved by a large, multi-subunit ubiquitin ligase, the anaphase promoting complex (APC/C), which induces degradation of mitotic cyclins and of the CDK-activating phosphatase Cdc25 (King et al., 1995; Donzelli et al., 2002; Teixeira and Reed, 2013). In vertebrates, the APC/C also targets the Cdt1 inhibitor geminin for degradation (McGarry and Kirschner, 1998).

In order to initiate S phase, two helicase coactivators – Cdc45 and the GINS complex – are recruited to pre-RCs, assembling the active replicative helicase, the CMG complex (Cdc45-Mcm2-7-GINS). Once the initial unwinding occurs, DNA polymerases and the sliding clamp, PCNA, are recruited to assemble the replisome and establish the replication fork (Leman and Noguchi, 2013). Origin firing requires a rise in CDK activity. Accordingly, APC/C activity is downregulated, mainly by inhibition of its regulatory subunit Cdh1 (Eldridge et al., 2006; Fukushima et al., 2013; Lau et al., 2013), but also by autoubiquitylation and degradation of its cognate ubiquitin conjugating enzyme (E2), UbcH10, a process induced in the absence of APC/C substrates (Rape and Kirschner, 2004). This allows an accumulation of G1-specific cyclins. Additionally, CDK inhibitors, such as p27 and p21, are degraded (Starostina and Kipreos, 2012). Three different E3s, KPC, Pirh2 and the Skp1-Cullin-F-box complex SCF^Skp2^, are known to act on p27 in a temporally and spatially ordered fashion during G1 and early S phase. The p21 protein is also a substrate of SCF^Skp2^, but in addition, this factor is targeted by an intriguing mechanism that directly couples ubiquitylation to S phase entry (Abbas et al., 2008; Kim et al., 2008). The cognate E3, Cullin-RING ligase CRL4^Cdt2^, recognizes its substrate only in conjunction with the replication clamp, PCNA, and only when PCNA is encircling DNA. The relevant degradation signal, which includes a PCNA-interacting peptide (PIP), is also found in other factors whose removal is associated with S phase, such as the fission yeast inhibitor of ribonucleotide reductase, Spd1, and the G1-specific transcription factor E2F1 from *Drosophila melanogaster*. (Havens and Walter, 2011; Ulrich, 2014). Thus, by coupling substrate recognition to binding of loaded PCNA, CRL4^Cdt2^ is able to read the state of the replication machinery as an activating signal.

Firing of origins needs to be strictly limited to once per cell cycle to avoid problems of re-replication. This is achieved through a process known as origin licensing that restricts pre-RC assembly to G1 (Moreno and Gambus, 2015). In order to render the process irreversible, essential loading factors, such as Cdt1 and Cdc6, are eliminated when cells enter S phase. In many organisms, this is again mediated by ubiquitin-mediated proteolysis. Human Cdt1 is ubiquitylated by at least two different E3s of the CRL family, SCF^Skp2^ and – as described above – CRL4^Cdt2^ (Li et al., 2003; Zhong et al., 2003). Cdc6 is deactivated either by export from the nucleus or by degradation following its ubiquitylation by CRL4^Cdt2^ (Saha et al., 1998; Clijsters and Wolthuis, 2014). In budding yeast, SCF^Cdc4^ mediates ubiquitylation of Cdc6 (Drury et al., 1997).

In contrast to the pervasive influence of ubiquitin, SUMO appears to exert more subtle regulatory effects on replication initiation. In a cell-free system based on *Xenopus laevis* egg extracts, inhibition of SUMOylation was found to increase replication rates by allowing a larger number of origins to fire (Bonne-Andrea et al., 2013). The negative effect of SUMO on origin firing was attributable to the modification of cyclin E following recruitment of the cyclin E-CDK complex to pre-RCs. The notion that most cells only use a sub-set of their potential origins in each S phase suggests that SUMO may in this context contribute to limiting excessive origin firing. In the budding yeast, Wei and Zhao (2016) recently reported an apparently unrelated phenomenon that likewise suggests a negative impact of SUMO on origin firing. They observed a cell-cycle regulated SUMOylation of Mcm2-7, peaking at the pre-RC stage when the complex is loaded onto origins, but declining upon origin firing at the G1-to-S transition. Artificial enhancement of local SUMOylation inhibited CMG assembly and origin firing, most likely by means of recruiting a phosphatase that reversed essential phosphorylation events required for CMG activation. Intriguingly, both SUMOylation and deSUMOylation of Mcm proteins are accomplished by multiple E3s and isopeptidases in a subunit-specific manner, and significant differences were noted in the cell cycle regulation of individual Mcm subunits (de Albuquerque et al., 2016). Moreover, it is important to note that other components of pre-RCs have also been identified as SUMOylation targets, among them the subunits of ORC (Golebiowski et al., 2009). Hence, it remains to be established whether the negative effect of SUMO on origin firing observed in this study is due to the modification of an individual Mcm subunit, the Mcm2-7 complex in its entirety, or a general accumulation of SUMO around the pre-RC.

### Proteomic Analyses of Replicating Chromatin

A wealth of information has emerged from the isolation of chromatin-associated proteins from proliferating cells, followed by mass spectrometry. Proteome-wide analyses identified numerous replication factors as ubiquitylation targets in human cells, including integral components of the replisome such as GINS and the Mcm2-7 helicase complex, the Replication Factor C (RFC) clamp loader complex, as well as all the replicative DNA polymerases and many associated factors (Wagner et al., 2011). Comparison of substrate spectra in the absence and presence of the proteasome inhibitor MG132 revealed both proteolytic and non-proteolytic roles of ubiquitylation. Similarly, a systematic screen in the budding yeast *Saccharomyces cerevisiae* identified a significant number of replisome components targeted by SUMO, including components of the Mcm2-7 complex, subunits of DNA polymerases and the RFC complex, the Rad27 flap endonuclease and topoisomerases Top1 and Top2 (Cremona et al., 2012). A recent study, using a procedure to isolate proteins on nascent DNA (iPOND) followed by mass spectrometry, characterized proteins enriched in the proximity of replisomes in an unprecedented spatial resolution. Interestingly, SUMOylation was predominant on factors near the replisome, while ubiquitylated proteins prevailed on mature chromatin (Lopez-Contreras et al., 2013). Although the implications of this distribution are not well understood, an appropriate balance appears to be important for replication and genome stability, as the ubiquitin isopeptidase USP7 was found to be responsible for maintaining SUMOylated proteins at replication forks by means of protecting them from ubiquitylation (Lecona et al., 2016). USP7 activity was found essential for origin firing as well as replisome progression, and intriguingly, one of its functions appears to be the deubiquitylation of SUMO itself.

Despite these observations, the functions of most replisome-associated modifications remain to be explored, and the notion that many of the SUMOylation events were found to be enriched after exposure of the cells to DNA damage (Cremona et al., 2012) raises the question of whether these modifications are inherent in the replication process or represent a response to spontaneous replication problems or low-level DNA damage.

### PCNA Modifications during Unperturbed DNA Replication

Posttranslational modifications heavily modulate the function of the eukaryotic sliding clamp. PCNA is a homotrimeric, ring-shaped complex that encircles DNA and functions as a processivity factor for DNA polymerases. In addition, PCNA serves as an interaction platform for numerous factors involved in DNA replication, repair, chromatin dynamics, cohesion and cell cycle regulation (Moldovan et al., 2007; Ulrich and Takahashi, 2013).

**FIGURE 1:**
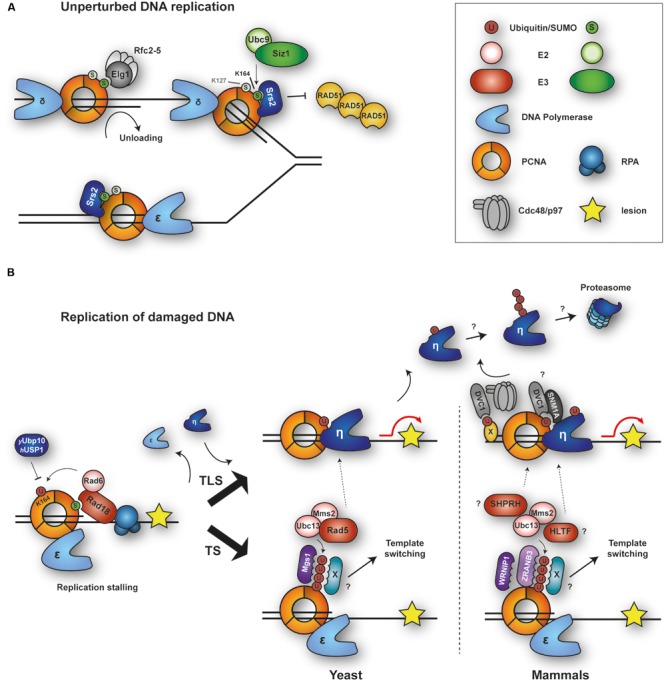
**Modifications of PCNA by ubiquitin and SUMO during replication of intact and damaged DNA. (A)** During unperturbed replication, budding yeast PCNA is SUMOylated at K164 and to a minor extent at K127. Modification at K164 is mediated by SUMO E2-E3 Ubc9-Siz1. PCNA^SUMO^ recruits anti-recombinogenic helicase Srs2 to counteract Rad51 filament formation. RFC-like complex RLC-Elg1 interacts with PCNA^SUMO^ and unloads PCNA from DNA. **(B)** Upon replication stalling and exposure of ssDNA, E2-E3 complex Rad6-Rad18 is recruited by interaction with the RPA complex and (in yeast) SUMO and monoubiquitylates PCNA at K164. This modification is removed by Ubp10 (yeast) or USP1 (humans). Monoubiquitylated PCNA recruits damage-tolerant DNA polymerases for translesion synthesis (TLS), while polyubiquitylated PCNA initiates template switching (TS) by a poorly defined mechanism. Auxiliary factors DVC1 and SNM1A modulate TLS. DVC1 cooperates with the AAA ATPase p97 to extract polymerase η from chromatin. Mgs1/WRNIP1 and ZRANB3 bind to polyubiquitylated PCNA and might contribute to TS. Ubiquitylation of TLS polymerases prevents association with PCNA^Ub^ and may induce their degradation.

During unperturbed replication, budding yeast PCNA is modified by SUMO at a highly conserved lysine, K164, and to a minor extent at K127 (Hoege et al., 2002; **Figure 1A**). Modification at K164 is mediated by the SUMO E2 Ubc9 in combination with the SUMO E3 Siz1 and is triggered by loading of the clamp onto DNA (Parker et al., 2008). SUMOylation at K127 *in vivo* requires Siz2 (Parker et al., 2008). The modification enhances interaction with an antirecombinogenic helicase, Srs2, at replication forks. Srs2 interacts with PCNA^SUMO^ via its carboxy-terminal tail containing a PIP-like PCNA interaction motif adjacent to a canonical SUMO interacting motif (SIM; Pfander et al., 2005; Armstrong et al., 2012). Recruitment of Srs2 prevents unwanted homologous recombination (HR) by disrupting Rad51 filaments (Krejci et al., 2003; Veaute et al., 2003; Papouli et al., 2005; Pfander et al., 2005). In addition, the presence of SUMO on PCNA boosts the damage-induced activity of the ubiquitin ligase Rad18 toward PCNA, again through a SIM in the E3 sequence (Parker and Ulrich, 2012). As a consequence, upon encounter of replication-stalling DNA lesions, damage processing is channeled into a bypass pathway that depends on PCNA ubiquitylation (**Figure 1B**, and see below). Hence, PCNA SUMOylation appears to function as a pre-emptive defense measure to influence pathway choice in response to replication stress. The modification also appears to enhance interaction with an alternative clamp loader complex, RLC-Elg1, which has been proposed to mediate PCNA unloading during replication (Parnas et al., 2010; Kubota et al., 2013). However, SUMOylation is not essential for Elg1 action on PCNA.

SUMOylation at K164 has been observed not only in budding yeast, but also in *X. laevis* egg extracts, chicken DT40 cells and, more recently, in mammalian cells (Leach and Michael, 2005; Arakawa et al., 2006; Gali et al., 2012; Moldovan et al., 2012). In human cells, expression of a PCNA-SUMO fusion protein inhibits spontaneous as well as damage-induced HR (Gali et al., 2012). Furthermore, a novel PCNA-interacting factor, the helicase PARI, has been suggested to function analogously to Srs2 in humans: it contains PIP and SIM motifs for interaction with PCNA^SUMO^ and suppresses HR by removing Rad51 from DNA (Moldovan et al., 2012). However, SUMOylated PCNA is present at very low levels in mammalian when compared to yeast cells, and its detection requires overexpression of epitope-tagged SUMO alleles (Gali et al., 2012). Whether this reflects the need for a tighter regulation of the process in the yeast system with its naturally higher rate of recombination remains to be explored.

In response to DNA damage, PCNA is mono- and polyubiquitylated at K164 (Hoege et al., 2002), which facilitates the bypass of replication-blocking lesions (see below). In fission yeast, however, these modifications are observed during S phase even in the absence of exogenous DNA-damaging agents (Frampton et al., 2006). Similarly, PCNA monoubiquitylation has been detected during replication of undamaged DNA in *X. laevis* egg extracts and was found to be required for efficient chromosomal replication (Leach and Michael, 2005). It is currently unclear, however, whether PCNA ubiquitylation contributes to the normal replication process itself or rather reflects higher levels of endogenous damage or fork problems in these systems.

### Modification of DNA Polymerases

All replicative DNA polymerases have been identified as ubi quitin and/or SUMO targets in budding yeast and mammalian cells (Wagner et al., 2011; Cremona et al., 2012). Mammalian DNA polymerase δ, responsible mainly for lagging strand synthesis, consists of four subunits (Hubscher et al., 2002), two of which, p12 and p66, are ubiquitylated during a normal S phase without leading to proteasomal degradation (Liu and Warbrick, 2006). Additionally, p66 is modified by SUMO at two different residues, K258 and K433 (Liu and Warbrick, 2006). Although the biological significance of these modifications remains unclear, it has been proposed that they might regulate protein–protein interactions within the polymerase complex or with other replication factors (Liu and Warbrick, 2006). A study in *Schizosaccharomyces pombe* showed that the catalytic subunit of the leading strand polymerase 𝜀, Pol2, is polyubiquitylated and undergoes significant proteasome-dependent degradation during unperturbed S phase, involving the ubiquitin ligase SCF^Pof3^ (the homolog of budding yeast SCF^Dia2^; Roseaulin et al., 2013). In contrast, Pol3, the catalytic subunit of polymerase δ, remained stable despite being ubiquitylated. The authors propose that the high rate of Pol2 turnover might ensure a continuous supply of “fresh” polymerase at the leading strand, while the discontinuous nature of lagging strand synthesis would not require an active exchange mechanism (Roseaulin et al., 2013). It will be interesting to address whether polymerase 𝜀 degradation serves a regulatory or a quality control purpose, and whether the phenomenon is conserved in other organisms.

### Modification of Mcm10

The essential, conserved minichromosome maintenance protein 10 (Mcm10) facilitates initiation of DNA replication. The protein is loaded onto replication origins at the G1/S transition, where it promotes strand separation either by activating the helicase or by stabilizing the formation of ssDNA, but it is dispensable for assembly of the helicase itself (Kanke et al., 2012; van Deursen et al., 2012; Thu and Bielinsky, 2013). A contribution of Mcm10 to the elongation step of DNA replication remains controversial (Thu and Bielinsky, 2013). Mcm10 has been shown to interact with the catalytic subunit of DNA polymerase α (Pol1) and regulate its stability, suggesting a role of Mcm10 in lagging strand synthesis (Ricke and Bielinsky, 2004). Ricke and Bielinsky (2004) reported that a small fraction of Mcm10 is monoubiquitylated at two distinct lysine residues during G1 and S phase of the cell cycle (Das-Bradoo et al., 2006). The modification promotes interaction with PCNA, but inhibits binding of Mcm10 to polymerase α. Moreover, mutations within Mcm10’s PIP box render cells inviable, suggesting that the interaction between Mcm10 and PCNA is essential (Das-Bradoo et al., 2006). Based on these findings, it was speculated that ubiquitylation of Mcm10 might induce a conformational change to expose its PIP box, thus allowing interaction with PCNA and release of polymerase α after the priming event. This might in turn facilitate the recruitment of polymerase δ and thereby Okazaki fragment extension (Das-Bradoo et al., 2006; Thu and Bielinsky, 2013).

### Replication Termination

Convergence of two replication forks leads to replication termination via disassembly of the replicative helicase. This process must be tightly controlled, as the CMG complex cannot be reloaded after initiation and must remain associated with the replication fork until completion of the replication unit, the replicon. However, in contrast to replication initiation, the mechanism of replisome disassembly is not well understood.

**FIGURE 2:**
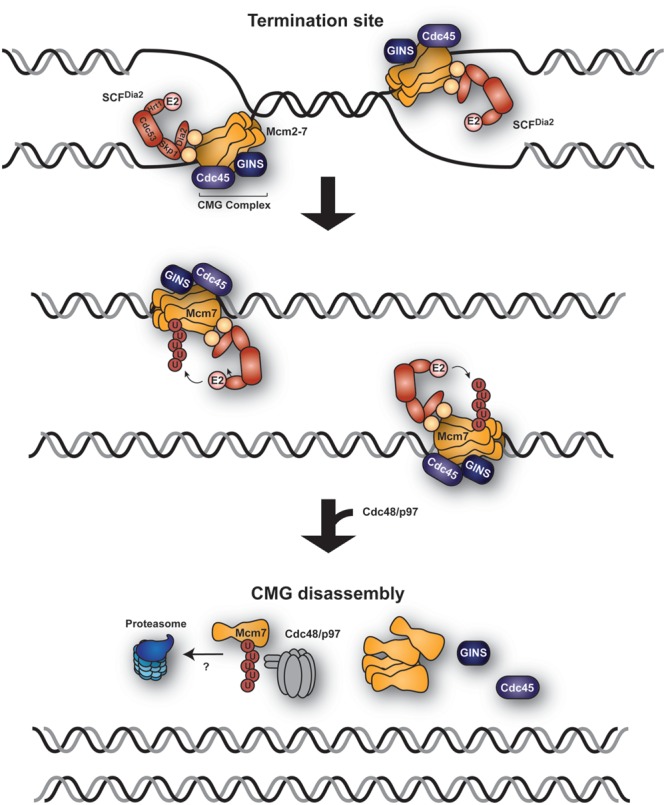
**Replication termination via ubiquitin-mediated CMG helicase extraction.** A model, derived from observations in budding yeast and *Xenopus laevis* egg extracts, proposes ubiquitylation of Mcm7 by the replisome-associated E3 SCF^Dia2^ at the sites of replication termination where two forks converge. The CMG helicase (Mcm2-7, Cdc45, and GINS) is subsequently extracted from the chromatin by Cdc48/p97 in a ubiquitin-dependent manner.

Two recent reports have helped to shed light on this reaction in budding yeast and *X. laevis* egg extracts, uncovering a key role for the ubiquitin system (**Figure 2**; Maric et al., 2014; Moreno et al., 2014). Helicase disassembly is triggered by K48-polyubiquitylation of the helicase subunit Mcm7 by a member of the SCF family of ubiquitin ligases. In budding yeast, the relevant F-box protein is Dia2 (Maric et al., 2014). Interestingly, SCF^Dia2^ had previously been identified as a component of the replication progression complex (RPC), tethered to the Ctf4 and Mrc1 subunits via a TPR domain within Dia2 (Mimura et al., 2009; Morohashi et al., 2009). SCF^Dia2^ was also proposed to mediate degradation of Ctf4 and Mrc1 (Mimura et al., 2009); however, a recent study has challenged this model and reported instead that SCF^Dia2^ tethering to the RPC is important for the efficient ubiquitylation of Mcm7 (Maculins et al., 2015). Inhibition of replication fork progression prevents Mcm7 ubiquitylation, suggesting that Mcm7 ubiquitylation is restricted to terminating replisomes (Maric et al., 2014; Moreno et al., 2014). How cells distinguish these from elongating complexes to avoid premature ubiquitylation and disassembly of the helicase is currently unknown. One possible scenario is that ubiquitylation is triggered by a DNA-mediated signal: while during replication the CMG helicase encircles ssDNA, it must enclose dsDNA upon termination (Bell, 2014). The mechanism of CMG helicase disassembly is likely to be conserved in higher eukaryotes. Homologs of Dia2 have yet to be identified, but other ubiquitin ligases might be involved in the process. Notably, disassembly of the CMG helicase requires the ubiquitin-dependent segregase p97, also called VCP (in yeast: Cdc48; Maric et al., 2014; Moreno et al., 2014), an AAA ATPase that remodels and thus extracts ubiquitylated proteins from protein complexes, membranes or chromatin, in many cases presenting them for proteasomal degradation (Vaz et al., 2013; Franz et al., 2016). Inactivation of p97 led to the accumulation of ubiquitylated forms of CMG on the chromatin, while inhibition of the proteasome did not block CMG disassembly (Maric et al., 2014; Moreno et al., 2014). Thus, whether Mcm7^Ub^ is degraded after extraction remains to be seen, although the K48-linkage of the polyubiquitin chain on Mcm7 would imply proteasomal action.

## Ubiquitin and SUMO in DNA Replication Stress

Replication stress is broadly defined as a condition that interferes with replication fork progression (Zeman and Cimprich, 2014). It is caused by a range of intrinsic or exogenous factors, including polymerase inhibition or nucleotide depletion, imbalances in the levels of replication proteins, interference from ongoing transcription, incorporation of ribonucleotides, or physical barriers to the DNA polymerases, such as sequences inherently prone to form secondary structures, tightly bound proteins, or DNA lesions arising from chemical alterations or strand breaks. Conditions that impair the replicative DNA polymerases without impeding strand unwinding by the helicase result in an accumulation of ssDNA. This in turn initiates a replication-specific checkpoint response via the protein kinase ATR in order to stabilize stalled replication intermediates, suppress the firing of late origins and prevent entry into mitosis (Jossen and Bermejo, 2013). Depending on the nature of the blockage, ATR signaling promotes replication fork rescue or restart in one of several ways, for example by means of re-priming downstream of the problematic region, fork reversal, translesion synthesis or strand exchange between the sister chromatids (Jossen and Bermejo, 2013; Leman and Noguchi, 2013). Prolonged replication fork stalling or lack of an appropriate checkpoint response can cause replication fork collapse. This poorly defined event may include a dissociation of the replisome and/or the formation of strand breaks, caused either passively or by the action of nucleases. Importantly, fork collapse triggers the transition to a genuine DNA damage response, mediated by the checkpoint kinase ATM, as is generally observed in response to DSBs. Over the past decade it has become clear that ubiquitin and SUMO are key regulators of both the replication- and the damage-associated branches of the checkpoint response (Ulrich, 2012; Jackson and Durocher, 2013).

### Proteomic Analyses of the DNA Replication Stress Response

A number of large-scale proteomic studies and systematic analyses of chromatin-associated factors have illustrated the dynamics of ubiquitylation and SUMOylation specifically in response to replication stress (Povlsen et al., 2012; Bursomanno et al., 2015; Xiao et al., 2015). According to those studies, when replication forks encounter DNA lesions, a plethora of SUMO and ubiquitin modifications on multiple factors is upregulated to either protect replication forks or initiate DNA repair mechanisms. In many cases, their consequences are mechanistically and functionally not well characterized, and it is clear today that modification of entire protein groups is sometimes more important than ubiquitylation or SUMOylation of individual factors (Psakhye and Jentsch, 2012). Moreover, a clear distinction between replication stress triggered by fork stalling and a full-blown damage response that might result from subsequent fork collapse has not always been attempted.

### Control of Homologous Recombination during DNA Replication

Homologous recombination serves as a means to repair DNA DSBs, to promote exchange of genetic material and proper chromosome segregation during meiotic cell divisions, and to rescue stalled or collapsed replication forks (Krejci et al., 2012). The process is initiated by strand breaks or – in particular at stalled replication forks – regions of ssDNA, tightly bound by the Replication Protein A (RPA) complex. RPA is exchanged for the recombination factor RAD51. In yeast, this step is promoted by the RAD52 protein. In human cells, the exchange is mainly mediated by BRCA2 (Jensen et al., 2010; Liu J. et al., 2010; Thorslund et al., 2010). The RAD51-ssDNA filament invades dsDNA, forming a so-called D-loop, and exchange of genetic material proceeds via a combination of DNA synthesis, branch migration and resolution or dissolution of recombination intermediates by the action of nucleases. Numerous auxiliary factors, among them DNA helicases and DNA-dependent ATPases, modulate HR activity either positively or negatively at every step (Krejci et al., 2012), and many of them are modulated in their activities by ubiquitin and/or SUMO.

#### Replication Protein A (RPA)

**FIGURE 3:**
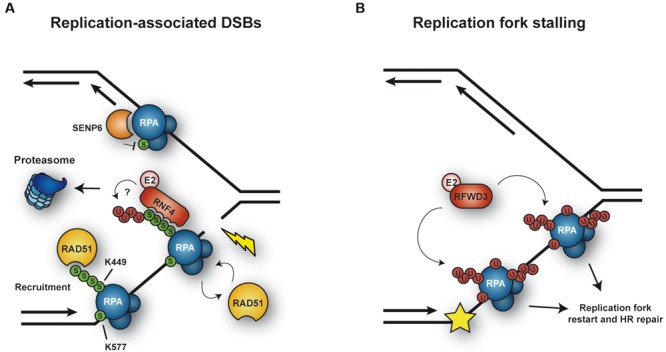
**Ubiquitylation and SUMOylation of RPA under conditions of replicative stress. (A)** SUMOylation of RPA1 is counteracted by the protease SENP6 during unperturbed S phase. In response to replication-mediated DSBs, SENP6 dissociates, allowing SUMOylation of RPA1 in order to facilitate HR via recruitment of RAD51. In addition, RPA^SUMO^ is recognized by the STUbL RNF4, which mediates proteasomal turnover of RPA1, thereby promoting exchange of RPA1 for RAD51. **(B)** Upon replication fork stalling, the ubiquitin E3 RFWD3 ubiquitylates RPA at multiple sites, thereby promoting replication fork restart and HR repair.

Replication protein A is a ssDNA-binding protein complex with a central role as a scaffold in virtually all DNA transactions. In eukaryotes, RPA consists of three subunits: RPA1, RPA2, and RPA3 (Zou et al., 2006). In mammals, the largest subunit, RPA1, is stably associated with the Sentrin/SUMO-specific protease SENP6 during S phase, which keeps RPA1 in a hypoSUMOylated state (Dou et al., 2010; **Figure 3A**). In response to replication-mediated or radiation-induced DSBs, SENP6 dissociates, resulting in modification of RPA1 with SUMO through the action of unknown SUMO ligases. Two lysine residues were identified as SUMO acceptor sites: K449 was modified by a poly-SUMO chain, whereas K577 was mono-SUMOylated. Importantly, treatment with hydroxyurea (HU) or UV irradiation, which stalls replication forks without causing DSBs, did not alter the association between SENP6 and RPA1. SUMOylation of RPA enhanced its interaction with RAD51 *in vitro* and promoted HR *in vivo* (**Figure 3A**). Taken together, RPA1^SUMO^ seems to facilitate recruitment of RAD51 to collapsed forks and DSBs, thereby initiating HR (Dou et al., 2010). Interestingly, RAD51 contains a SIM motif that is necessary for its accumulation at damage sites (Shima et al., 2013). However, whether RPA1^SUMO^ is indeed the *in vivo* target of this SIM relevant for recruitment of RAD51 to damage sites needs to be demonstrated, considering that SUMOylation of other proteins might act synergistically or redundantly in the assembly of repair complexes (Psakhye and Jentsch, 2012). The yeast homolog of RPA1, Rfa1, is also modified by SUMO upon treatment with the alkylating agent methyl-methanesulfonate (MMS; Burgess et al., 2007; Cremona et al., 2012), although the functional significance of this modification remains unclear.

More recently, Galanty et al. (2012) posited a plausible mechanism for the transition from RPA to RAD51on ssDNA, relying on the SUMO-targeted ubiquitin ligase (STUbL) RNF4: in RNF4-depleted cells RAD51 fails to accumulate and RPA persists at lesions. A SUMOylation-defective RPA1 mutant exhibited a similar behavior. Based on these findings, RNF4 was proposed to target RPA1^SUMO^ for proteasomal degradation (**Figure 3A**). Consistent with this model, RNF4 and RPA1 coimmunoprecipitated in a manner dependent on the SIM region of RNF4, suggesting that RNF4 directly recognizes RPA1^SUMO^ as a ubiquitylation target. As a consequence, RPA1 accumulates in RNF4-depleted cells after exposure to DNA damage, and several proteasome subunits become detectable at damage sites in an RNF4-dependent manner. Thus, RNF4-mediated RPA1 turnover might promote the exchange of RPA1 for RAD51 on ssDNA, stimulating HR (Galanty et al., 2012). This mechanism and the recruitment of RAD51 through RPA^SUMO^ are not mutually exclusive, as both could cooperate in promoting RAD51 filament formation (**Figure 3A**). However, the direct ubiquitylation of SUMO-modified RPA1 by RNF4 has yet to be demonstrated.

On the other hand, RPA is also ubiquitylated under conditions complementary to those that trigger its SUMOylation (**Figure 3B**). Ubiquitylation on multiple sites of all three RPA subunits was observed in response to replication fork stalling upon UV irradiation or treatment with other fork-stalling agents such as 4-nitroquinoline oxide or HU, but not after exposure to ionizing radiation (Elia et al., 2015). Thus, apparently cells respond to different types of damage in distinct ways, either ubiquitylating or SUMOylating RPA. Ubiquitylation of RPA, mediated by the E3 RFWD3, does not lead to proteasomal degradation. Inhibition of the modification by RFWD3 depletion or by means of a ubiquitylation-deficient RPA mutant caused defects in fork restart and persistence of γ-H2AX foci after release from prolonged HU treatment, as well as a reduction in HR in response to both fork stalling and direct induction of DSBs. These findings imply that RFWD3-dependent ubiquitylation of RPA promotes fork stability and HR-mediated restart of collapsed forks upon exposure to replication stress (Elia et al., 2015). The exact mechanism by which RPA^Ub^ stimulates these effects remains elusive, even though it has been suggested that the modification may promote release of the RPA complex from DNA and/or facilitate the recruitment of HR factors, similar to RPA^SUMO^ (Elia et al., 2015). Whether ubiquitin and SUMO can coexist on the same RPA complex remains to be explored. An independent study identified another ubiquitin ligase, PRP19, as the E3 responsible for RPA ubiquitylation (Marechal et al., 2014). In this study, depletion of PRP19 was found to reduce damage-induced RPA ubiquitylation and compromise the accumulation of the ATR-ATRIP checkpoint complex at sites of damage. In addition, ATRIP was reported to exhibit an affinity for K63-linked ubiquitin chains, suggesting that this modification on RPA might contribute to the recruitment of ATR-ATRIP (Marechal et al., 2014). However, these findings have been called into question by the observation of an unintentional side effect of the siRNAs used for the depletion of PRP19, on exogenously expressed ubiquitin (Elia et al., 2015). Hence, an involvement of PRP19 in RPA ubiquitylation needs to be reconfirmed.

#### BLM

**FIGURE 4:**
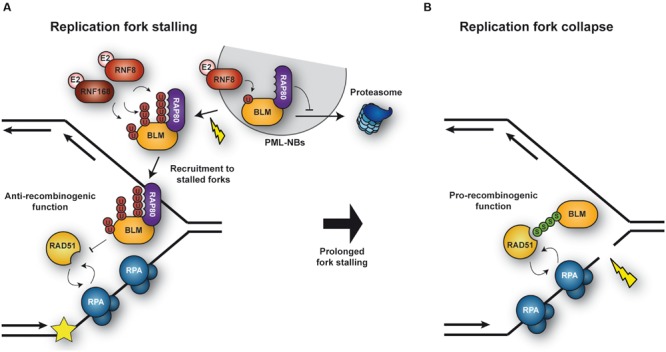
**Regulation of BLM activity under conditions of replicative stress. (A)** Upon replication fork stalling, BLM is polyubiquitylated by the ubiquitn E3s RNF8 and RNF168. Polyubiquitylated BLM is recognized by RAP80, which mediates relocation of BLM from PML nuclear bodies and recruitment to stalled forks. At the fork BLM suppresses unwanted HR events. **(B)** Upon collapse of a stalled fork, BLM is SUMOylated, thereby facilitating the recruitment of RAD51 and repair by HR.

The RecQ DNA helicase BLM plays an important role in genome maintenance by facilitating HR-mediated DNA repair in various ways (Bohm and Bernstein, 2014). BLM protein levels are regulated during the cell cycle, being lowest in G1 and peaking in late S phase (Dutertre et al., 2000). BLM normally resides in promyelocytic leukaemia (PML) nuclear bodies but re-localizes to stalled replication forks in response to DNA damage (Sengupta et al., 2003). At replication forks, BLM can exert both pro- and anti-recombinogenic functions (**Figure 4**): it protects replication forks by suppressing the formation of aberrant recombination events or, upon fork collapse, it promotes repair by HR. Posttranslational modifications of BLM by ubiquitin and/or SUMO make key contributions to the regulation of these processes (Bohm and Bernstein, 2014). Monoubiquitylation of BLM in the absence of DNA damage appears to be important for its normal localization in PML nuclear bodies (**Figure 4A**). Following HU treatment, BLM is further polyubiquitylated with K63-linked chains at K105, K225, and K259 by the E3s RNF8 and RNF168. Polyubiquitylation of BLM was found to be required for its recruitment to stalled replication forks, mediated via interaction with the ubiquitin-interacting motifs of the adaptor protein RAP80 (Tikoo et al., 2013). Once at stalled replication forks, BLM suppresses excessive HR (Tikoo et al., 2013) by dismantling RAD51-ssDNA filaments and disrupting D-loops (Bugreev et al., 2007). Polyubiquitylation of BLM might also potentiate the protein’s anti-recombinogenic effect. However, constitutive association of BLM with chromatin, achieved by fusion with histone H2AX or the FHA domain of MDC1, was sufficient to suppress the elevated levels of HR caused by depletion of either RNF8 or RNF168, indicating that polyubiquitylation of BLM might function more as a means to recruit rather than to activate the protein (Tikoo et al., 2013).

In addition to being ubiquitylated, BLM is modified by SUMO at multiple sites, preferentially at K317 and K331 (Eladad et al., 2005). Expression of a SUMOylation-defective BLM mutant induces an excess of γ-H2AX foci, DSBs and cell death under conditions of replication stress, such as prolonged HU treatment, uncovering a role of BLM SUMOylation in protecting and/or restarting replication forks (**Figure 4B**). Interestingly, cells unable to SUMOylate BLM also fail to recruit RAD51 and to induce HR at stalled replication forks (Ouyang et al., 2009). In fact, as described for RPA (Dou et al., 2010), SUMOylation of the helicase enhances binding to RAD51 *in vitro* (Ouyang et al., 2009). However, in contrast to its ubiquitylation, its SUMOylation was not required for the trafficking of BLM itself to stalled forks (Ouyang et al., 2009). Thus, BLM SUMOylation might function as a molecular switch to regulate its activity: unSUMOylated, polyubiquitylated BLM is recruited to stalled replication forks, protecting them from deleterious HR, while BLM^SUMO^ facilitates HR by promoting RAD51 recruitment to collapsed forks (**Figure 3**; Ouyang et al., 2009). Future studies will certainly provide insight into the molecular mechanism by which these modifications regulate BLM function.

Little is known about posttranslational modifications of the BLM ortholog in budding yeast, Sgs1. In the absence of Sgs1, cells accumulate Rad51-dependent cruciform structures at damaged replication forks (Liberi et al., 2005). The same is observed in mutants of the SUMO-conjugating enzyme, *ubc9* (Branzei et al., 2006), and interestingly, Sgs1 is indeed a target of SUMOylation, suggesting the possibility that the modification might be important to prevent the accumulation of aberrant recombinogenic structures during replication of damaged templates (Branzei et al., 2006). However, in contrast to BLM modification, SUMOylation of Sgs1 does not seem to influence recombination frequencies (Lu et al., 2010).

Sgs1 modifications also appear to impinge on the protein’s subcellular localization (Bohm et al., 2015): During S phase, Sgs1 forms nuclear foci that likely indicate spontaneous recombination events, as they increase with ionizing radiation treatment. Upon replication fork stalling by nucleotide depletion, the number of these foci is strongly reduced in a manner depending on the STUbL Slx5/8 – suggesting that STUbL-mediated ubiquitylation contributes to removing Sgs1 from stalled forks, thus possibly preventing unwanted recombination. However, as overall Sgs1 levels do not decrease, the process does not appear to involve degradation of the helicase, but rather its re-localization. The mechanism is likely conserved, since BLM^SUMO^ is also targeted by the mammalian STUbL RNF4 (Galanty et al., 2012). Thus, SUMOylation of BLM/Sgs1 seems essential for the fine-tuning of the protein’s function: it facilitates HR repair at collapsed forks, but it also induces removal of the protein from stalled forks, adding an additional level of regulation.

#### SRS2

A recent study by Urulangodi et al. (2015) has uncovered a new mechanism promoting local recombination at sites of compromised replication in budding yeast. As described above, PCNA^SUMO^ recruits the helicase Srs2 to prevent unwanted recombination during unperturbed S phase. Hence, removal of Srs2 should be critical in order to engage HR after fork stalling. Urulangodi et al. (2015) identified Esc2, a protein containing two SUMO-like domains (SLDs), as a new factor associated with stalled replication forks and controlling Srs2 levels. Via its SLDs, Esc2 interacts with the SIM of Srs2, thereby promoting interaction of Srs2 with the STUbL complex Slx5/8 and subsequent degradation by the proteasome. Consistent with these findings, Srs2 SUMOylation is induced by DNA damage (Saponaro et al., 2010). Thus, local down-regulation of Srs2 appears to enable recruitment of Rad51 and thereby HR-mediated rescue of stalled forks (Urulangodi et al., 2015). In addition, it has been shown *in vitro* that Srs2 SUMOylation and interaction with PCNA^SUMO^ are mutually inhibitory (Kolesar et al., 2012), suggesting that Esc2 might help to dismantle the association between PCNA^SUMO^ and Srs2. Upon dissociation from PCNA^SUMO^, Srs2 would be free to undergo SUMOylation, which would disfavor re-association with PCNA^SUMO^. Alternatively, Esc2 might bypass the need for Srs2 SUMOylation by acting as a platform to recruit Slx5/8 to its substrate via physical interaction of Esc2 with Slx5 (Urulangodi et al., 2015).

#### SLX4

The binding of a multitasking protein to either SUMO or ubiquitin can modulate its function by conveying different contextual specificities. An example is provided by the scaffold protein SLX4, which coordinates multiple DNA repair pathways through its ability to bind several nucleases. Human SLX4 contains two ubiquitin-binding zinc finger (UBZ) domains that are essential for its role in the FA pathway, facilitating repair of DNA interstrand crosslinks (ICLs; see below and Coleman and Huang, 2016). In addition, SLX4 contains a cluster of SIMs, which recognizes SUMO chains. How much this cluster contributes to the repair of ICLs is not entirely clear, as expression of a SIM-defective mutant of SLX4 was able to effectively rescue the mitomycin C (MMC) sensitivity of SLX4-deficient human cells (Guervilly et al., 2015; Ouyang et al., 2015), whereas rescue was only partial in mouse cells (Gonzalez-Prieto et al., 2015). However, the SIM cluster is exclusively required for efficient recruitment and retention of SLX4 to laser-induced DNA damage sites, where it might enhance the association of SLX4 with multiple SUMOylated targets, including RPA and MRN (Gonzalez-Prieto et al., 2015; Ouyang et al., 2015). Once at stalled forks, SLX4 might, for instance, promote replication fork restart when associated with the endonuclease MUS81 (Hanada et al., 2007).

Interestingly, the SIMs perform yet another, unexpected function: by interacting with SUMO-charged Ubc9, they promote the SUMOylation of SLX4 itself and its binding partner, XPF. This presumed SUMO ligase activity appears to be toxic under some conditions, as mild overexpression of SLX4, but not mutation of the SIM or BTB domain, sensitizes cells to replication fork stalling upon HU treatment and promotes DSBs. In contrast, E3 activity was found to be required to prevent mitotic catastrophe at chromosome fragile sites, suggesting that promotion of DSB formation might be beneficial in difficult-to-replicate regions of the genome (Guervilly et al., 2015). Additional work will be needed to understand how the SUMO ligase activity of SLX4 contributes to genome stability and whether it can target other substrates besides SLX4 and XPF.

#### Structural Maintenance of Chromosomes 5 and 6 (Smc5/6)

**FIGURE 5:**
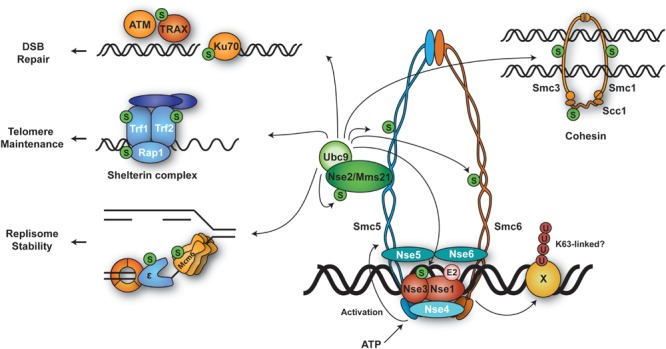
**Ubiquitylation and SUMOylation targets of Smc5/6 and their associated processes.** The Smc5/6 complex harbors SUMO ligase (Nse2) and ubiquitin ligase (Nse1-Nse3) subunits. DNA binding stimulates the ATPase domains at the globular heads of Smc5/6, inducing a conformational change in the coiled-coil region of Smc5 that activates the SUMO E3 activity of Nse2. Smc5/6 likely selects its ubiquitylation and/or SUMOylation targets at relevant loci where the complex is recruited.

The structural maintenance of chromosomes 5 and 6 (Smc5/6) complex (**Figure 5**) belongs to a family of multisubunit ATPases that also includes cohesion and condensin. The complex consists of eight subunits, Smc5, Smc6 and six non-Smc element (Nse) subunits, Nse1– 6 (Murray and Carr, 2008). Smc5 and Smc6 adopt extended coiled-coil structures with globular heads at the C- and N-termini that form an ATPase domain. It is believed that Smc5/6, like the related cohesin and condensin complexes, is able to embrace DNA double-strands and thereby influence higher chromatin organization. Consistent with this idea, Smc5/6 has been shown to sequester sister chromatid intertwinings and assist replication fork rotation to relieve super-helical tension generated as DNA unwinds ahead of the fork (Kegel et al., 2011).

In most organisms, all subunits of the Smc5/6 complex are essential for cell survival. Hypomorphic mutants of Smc5/6 show mark sensitivity to perturbation of replication such as reduced dNTP levels and DNA damage (Murray and Carr, 2008; Stephan et al., 2011). Moreover, Smc5/6 localizes to natural replication pausing sites such as rDNA, centromeres and telomeres, and to collapsed forks (Ampatzidou et al., 2006; Lindroos et al., 2006; Menolfi et al., 2015). It has been shown that HR intermediates such as X-shaped molecules accumulate in Smc5/6 mutants in the course of replication in yeast. This leads to lethality in mitosis due to failure of these mutants to properly segregate their chromosomes (Ampatzidou et al., 2006; Branzei et al., 2006; Chen et al., 2009; Irmisch et al., 2009; Bermudez-Lopez et al., 2010; Choi et al., 2010). Interestingly, restricting Smc5/6-activity to G2, i.e., after completion of genome replication, is compatible with survival (Bermudez-Lopez et al., 2010; Menolfi et al., 2015). These data suggest that Smc5/6 is important for resolving recombination structures formed during DNA replication.

One of the Smc5/6 subunits, Nse2, also known as Mms21, is known to be a SUMO ligase (Andrews et al., 2005; Potts and Yu, 2005; Zhao and Blobel, 2005), whereas Nse1 was proposed and subsequently shown to harbor ubiquitin ligase activity (Pebernard et al., 2008; Doyle et al., 2010).

##### Nse2/Mms21, a SUMO ligase associated with the Smc5/6 complex

Nse2 associates with the coiled-coil domain of Smc5 via an essential N-terminal domain. In contrast, its C-terminal SUMO E3 domain is dispensable for survival, but important for resistance to DNA damage (McDonald et al., 2003; Andrews et al., 2005; Potts and Yu, 2005; Zhao and Blobel, 2005; Duan et al., 2009). Cells lacking the Nse2 SUMO E3 activity accumulate recombination intermediates following DNA replication stress, similar to *smc5/6* hypomorphic mutants (Branzei et al., 2006; Chavez et al., 2010). This suggests that the Smc5/6 complex responds to DNA damage primarily through its associated SUMOylation activity.

A recent study has provided new insight into the activation mechanism of Nse2’s SUMO E3 activity toward its mostly chromatin-bound targets. Bermudez-Lopez et al. (2015) reported that ATP binding by the globular head of Smc5 induces a conformational change in the coiled-coil region, which was found to enhance E3 activity of Nse2. This mechanism appears to couple the loading of Smc5/6 onto chromatin to the activation of its enzymatic activity and suggests that the Smc5/6 complex as a whole behaves like a giant SUMO E3. In contrast to the SUMO ligases of the PIAS family, Nse2 lacks a DNA-binding domain (Jackson, 2001; Ulrich, 2014). Therefore, loading of the entire Smc5/6 complex is likely required for selecting chromatin-associated substrates. In fact, many of Nse2’s targets have been found to co-localize with Smc5/6 or with its associated repair sites. Not surprisingly, Nse2 SUMOylates several subunits within the Smc5/6 complex, such as Smc5, Smc6, Nse3, and Nse2 itself (Andrews et al., 2005; Potts and Yu, 2005; Zhao and Blobel, 2005). Interestingly, SUMOylation by the Smc5/6 complex impinges on the structurally related cohesin complex: in response to DNA damage Nse2 SUMOylates all cohesin subunits, Smc1, Smc3, and Scc1. The modification is required for proper loading of the cohesin complex under these conditions. Abolishing SUMOylation of cohesin by point mutations or by tethering a SUMO-specific isopeptidase to the complex caused defects in the establishment of sister chromatid cohesion and impaired cellular survival (Almedawar et al., 2012; Wu et al., 2012). Other substrates include DNA repair factors such as Ku70 and TRAX (Potts and Yu, 2005; Zhao and Blobel, 2005). In human cells, the complex modifies telomere-binding proteins like RAP1, TRF1, and TRF2 (Potts and Yu, 2007). In budding yeast, rDNA-associated proteins such as RNA polymerase I, Fob1, and Tof2, and the replication factors Pol2 and Mcm6 have been identified as substrates (Albuquerque et al., 2013; Hang et al., 2015). The functional consequences of these SUMOylation events are yet to be clarified.

##### Nse1, an Smc5/6 subunit with ubiquitin ligase activity

The Nse1 subunit of the Smc5/6 complex, a RING finger protein, exhibits weak ubiquitin ligase activity on its own (Pebernard et al., 2008). This activity is significantly enhanced in the presence of its direct interaction partner, Nse3. In collaboration with the E2 Ubc13/Mms2, Nse1/3 is capable of assembling K63-linked ubiquitin chains (Doyle et al., 2010). In *S. pombe*, the RING-like motif of Nse1 is not essential, but inactivation of the domain leads to hypersensitivity toward genotoxic stress (Pebernard et al., 2008). Recently, Nse3 was found to harbor DNA binding activity, and mutations in the relevant domain caused damage sensitivity and chromosome aberrations (Zabrady et al., 2015). These data indicate that Nse1/3 contribute to the activity of the Smc5/6 complex in chromosome maintenance upon genotoxic stress. However, the targets of such ubiquitin ligase activity have not been identified.

### DNA Damage Bypass

DNA damage bypass, also called DNA damage tolerance, is important in situations where fork stalling has been triggered by lesions in the replication template that cannot be copied by the replicative DNA polymerases (Saugar et al., 2014). Such lesions mostly represent damage that is subject to base or nucleotide excision repair, i.e., small or bulky adducts, oxidative lesions, abasic sites and UV-induced pyrimidine dimers. In order to prevent a permanent replication arrest, damage bypass ensures complete duplication of the affected region without actually removing the lesion, and excision-based repair can act subsequently when the DNA has regained its double-stranded form. Two major pathways of damage bypass can be distinguished, which differ significantly in their overall accuracy: on the one hand, specialized damage-tolerant DNA polymerases can copy damaged DNA in a process named translesion synthesis (TLS). Due to the low fidelity of the enzymes involved, this pathway is a major cause of damage-induced mutagenesis. On the other hand, error-free damage bypass can be accomplished by means of a so-called template switching (TS) pathway, which altogether avoids the use of the damaged DNA as a replication template and instead relies on the (undamaged) sister chromatid to provide accurate sequence information. This process involves recombination factors and joint molecules as intermediates (Giannattasio et al., 2014), but appears to be distinct from the classical HR mechanism used for DSB repair. Both branches of damage bypass can act in a postreplicative manner; thus, they are not necessarily coupled to replication fork progression (Daigaku et al., 2010; Karras and Jentsch, 2010).

#### Mono- and Polyubiquitylation of PCNA

The profound impact of damage bypass on replication efficiency and fidelity is reflected by an intricate regulation of the pathway in cells (Ulrich, 2009; McIntyre and Woodgate, 2015). Central to its activation is the ubiquitylation of PCNA on a conserved lysine residue, K164 (**Figure 1B**). Whereas monoubiquitylation by the E2-E3 pair Rad6-Rad18 promotes TLS, extension of the modification to a K63-linked polyubiquitin chain by the heterodimeric E2 Ubc13-Mms2 triggers error-free TS (Hoege et al., 2002; Stelter and Ulrich, 2003). The cognate E3 in budding yeast is the RING finger protein Rad5; its human homologs are HLTF and SHPRH (Motegi et al., 2008). Rad18, which is rate-limiting for both TLS and TS, is recruited by RPA-covered ssDNA through physical interactions with the RPA complex (Davies et al., 2008; Niimi et al., 2008). In budding yeast, damage-independent SUMOylation of PCNA (see above) provides a second signal that strongly stimulates Rad18’s activity toward PCNA (Parker and Ulrich, 2012). Additional E3s have been reported to operate on mammalian PCNA, such as RNF8 and CRL4^Cdt2^ (Simpson et al., 2006; Zhang et al., 2008; Terai et al., 2010; Krijger et al., 2011). Moreover, large-scale mass spectrometry studies have identified multiple other ubiquitylation sites (McIntyre and Woodgate, 2015). However, the relevance of these conjugation factors and modifications for damage bypass is still a matter of debate, and links to proteasomal degradation may not be excluded (Yu et al., 2009; Cazzalini et al., 2014).

Activation of TLS by monoubiquitylated PCNA can largely be explained by the presence of ubiquitin-binding domains within the major family of damage-tolerant polymerases, which convey an enhanced affinity for the modified form of PCNA (Watanabe et al., 2004; Bienko et al., 2005; Bi et al., 2006; Plosky et al., 2006). In mammals, direct interactions with Rad18 also contribute to the recruitment of TLS polymerases (Watanabe et al., 2004). Whereas in yeast TLS-mediated damage-induced mutagenesis nearly completely depends on PCNA ubiquitylation, the process appears to be less dependent on this modification in vertebrate cells (Stelter and Ulrich, 2003; Edmunds et al., 2008; Hendel et al., 2011). In addition to the damage-tolerant polymerases, a number of auxiliary factors have been proposed to modulate TLS via recognition of monoubiquitylated PCNA in mammals. These include the UBZ domain-containing proteins SNM1A, a nuclease that might provide a link between TLS and the repair of ICLs, and DVC1 (also called Spartan), an adaptor for the ubiquitin-dependent chaperone p97 (Yang et al., 2010; Centore et al., 2012). The selectivity of DVC1’s UBZ domain for PCNA^Ub^ has been contested, however, and it has been proposed that the protein binds to other ubiquitylated proteins at sites of replication stalling, where it would mediate extraction of polymerase η in order to limit TLS activity (Davis et al., 2012; Mosbech et al., 2012). Downregulation of TLS appears to be important for preventing excessive mutagenesis during replication. In human cells, this is accomplished mainly by PCNA deubiquitylation via the isopeptidase USP1 (Huang et al., 2006). In addition, the ubiquitin-like modifier ISG15 was recently found to contribute to the termination of TLS by modification of PCNA, which in turn mediated the recruitment of USP10 for PCNA deubiquitylation and dissociation of polymerase η (Park et al., 2014). Intriguingly, a viral isopeptidase, BPLF1, was also shown to deubiquitylate human PCNA during replication of the Epstein–Barr genome, thus inhibiting polymerase η recruitment during the lytic phase of infection (Whitehurst et al., 2012). How an inhibition of TLS may promote viral replication is not yet understood. In yeast, PCNA deubiquitylation is mediated by Ubp10; however, despite an accumulation of PCNA^Ub^, inactivation of the enzyme does not cause a noticeable increase in mutation rates, indicating that reversal of the modification may be less critical for damage bypass in this organism (Gallego-Sanchez et al., 2012).

How polyubiquitylation of PCNA triggers TS is still an unresolved question. From experiments using linear head-to-tail fusions of ubiquitin moieties as mimics of polyubiquitin chains it was inferred that the K63-linkage itself is important for TS activity (Zhao and Ulrich, 2010). Although putative effectors that preferentially interact with polyubiquitylated PCNA have been identified, they are unlikely to be directly responsible for activating TS: the human ATPase WRNIP1 and its yeast homolog Mgs1 accumulate at stalled replication intermediates in a manner that depends on their UBZ domain as well as PCNA ubiquitylation (Crosetto et al., 2008; Saugar et al., 2012). However, even though a subset of the phenotypes of *mgs1* mutants is consistent with a function downstream of PCNA^Ub^ (Hishida et al., 2006; Saugar et al., 2012), no obvious TS defects are observed in such mutants. In human cells, WRNIP1 appears to contribute to checkpoint activation as a bridging factor that promotes interaction of PCNA^Ub^ with the ATM-associated ATMIN protein (Kanu et al., 2015). Yet, this function is unlikely to be related to polyubiquitylation, as a single ubiquitin moiety is sufficient to stimulate the interaction between WRNIP1/Mgs1 with PCNA (Saugar et al., 2012). A second ATPase, ZRANB3, has also been implicated in PCNA-dependent damage bypass, based on its localization to laser-induced DNA damage, its preferential interaction with polyubiquitylated PCNA, and a general sensitivity to replication stress upon depletion of the protein (Ciccia et al., 2012; Weston et al., 2012). However, a function in the TS pathway has yet to be properly established by means of genetic analysis. Moreover, a convincing yeast homolog has not been identified, which argues for an auxiliary function of ZRANB3 rather than a key role in activating TS.

Analysis of the TS pathway is further complicated by the multi-functionality of Rad5 and its two human homologs, whose catalytic RING domains are embedded in SWI/SNF-like domains with helicase and DNA-dependent ATPase activity. Although this helicase function can be genetically separated from Rad5’s role in ubiquitin-dependent TS, it does contribute to survival of replication stress (Choi et al., 2015). Interestingly, Rad5 and its homologs have been implicated not only in TS, but also in TLS in budding and fission yeast as well as humans (Minesinger and Jinks-Robertson, 2005; Gangavarapu et al., 2006; Pages et al., 2008; Coulon et al., 2010; Lin et al., 2011; Kuang et al., 2013; Xu et al., 2016). Mechanistically, this activity remains controversial, as some studies have invoked PCNA polyubiquitylation in the process, whereas others have reported a RING- and ATPase-independent function or a dependence on a physical interaction with the TLS polymerase Rev1. Moreover, although both HLTF and SHPRH are capable of polyubiquitylating PCNA *in vitro*, they have been postulated to fulfill non-redundant functions in cooperation with TLS polymerases η and κ, respectively, depending on the nature of the damaging agent (Lin et al., 2011). Based on these observations, Lin et al. (2011) have put forth a model where a damage-tolerant polymerase harboring multiple UBDs, such as polymerase κ, might preferentially recognize polyubiquitylated PCNA, while monoubiquitylation might stimulate those polymerases with only one UBD, such as polymerase η. Along similar lines, Fuchs and coworkers proposed that polyubiquitylated PCNA might serve to simultaneously attract several different TLS polymerases for cooperation in damage bypass (Coulon et al., 2010). In contrast, observations by Yang et al. (2014) in an *in vitro* set-up have led to the opposite conclusion: rather than promoting TLS, polyubiquitylation of PCNA was found to inhibit the activity of polymerase η in the bypass of an abasic site, suggesting that the K63-chains trap the polymerase in a non-productive mode. In budding yeast, genetic analysis supports a positive effect of Rad5 on TLS in some situations. At the same time, however, PCNA polyubiquitylation promotes damage resistance even in the absence of any damage-tolerant polymerase, thus clearly implying a TLS-independent function in TS (Zhao and Ulrich, 2010). In summary, the consequences of PCNA polyubiquitylation remain to be elucidated in molecular terms, and future studies will be needed in order to gain insight into how the balance between mutagenic TLS and error-free TS is controlled *in vivo*.

#### Ubiquitylation of Other Damage Bypass Factors

Besides PCNA, numerous other factors involved in DNA damage bypass have been identified as ubiquitylation and/or SUMOylation targets (McIntyre and Woodgate, 2015). As many of the modifications were detected in the context of large-scale proteomics screens, their relevance for damage bypass has not always been confirmed. Nevertheless, some common patterns indicate potential regulatory impacts. For example, all human TLS polymerases of the Y-family are ubiquitylated, and in many cases this depends on their own ubiquitin-binding domains. Although this is reminiscent of the E3-independent phenomenon of coupled ubiquitylation (Hoeller et al., 2007), relevant ubiquitin ligases have actually been identified, such as Pirh2, Mdm2, and TRIP in the case of polymerase η (Jung et al., 2010, 2011, 2012; Wallace et al., 2014). While Pirh2 attaches monoubiquitin, which apparently inhibits TLS by preventing interaction of the polymerase with PCNA^Ub^ (Bienko et al., 2010), Mdm2 achieves the same effect by targeting polymerase η for polyubiquitylation and proteasomal degradation. In contrast, polyubiquitylation by the TRAF-interacting protein TRIP was reported to promote polymerase η localization to nuclear foci. The *D. melanogaster* homolog of TRIP, NOPO, is known to assemble K63-linked chains, possibly indicating a regulatory function of TRIP-mediated polymerase η modification as well, and interactions of both TRIP and NOPO with several Y-family polymerases suggest a conservation of the process (Wallace et al., 2014). In budding yeast, polymerase η has been found to be ubiquitylated as well; however, the effects of this modification on protein stability remain controversial (Parker et al., 2007; Skoneczna et al., 2007; Pabla et al., 2008; Plachta et al., 2015). Ubiquitin-mediated proteolysis also controls the levels of budding yeast TLS polymerase Rev1 along the cell cycle, thus limiting the bulk of its mutagenic activity to the G2/M phase (Waters and Walker, 2006). Finally, McIntyre et al. (2013) showed that ubiquitylation of human Y-family polymerases appears to promote mutual interactions via their ubiquitin-binding domains and – as a consequence – facilitate their cooperation in TLS.

Another recurring theme in the regulation of damage bypass is the protection of critical factors from proteolysis. This may be achieved by the SUMOylation of a protein, as is observed in the nematode *Caenorhabditis elegans*, where SUMOylation of polymerase η and potentially κ prevents their ubiquitin-mediated degradation during early embryonic development (Kim and Michael, 2008; Roerink et al., 2012). Alternatively, deubiquitylation can effectively stabilize a chromatin-associated protein by preventing its extraction or proteasomal degradation. In human cells, the isopeptidase USP7 appears to play a major role in this manner. As described above, its activity is important even during unperturbed replication (Lecona et al., 2016). In response to replication stress, USP7 deubiquitylates a number of proteins, among them polymerase η, Mdm2, Rad18, and HLTF. By acting on polymerase η and Mdm2, USP7 directly and indirectly stabilizes the polymerase and thereby facilitates TLS (Qian et al., 2015). Deubiquitylation of Rad18 and HLTF was also observed to stabilize the E3s and thus contribute positively to PCNA mono- and polyubiquitylation, respectively (Qing et al., 2011; Zlatanou et al., 2016). In addition, Zeman et al. (2014) reported that a failure to deubiquitylate Rad18 prevented its efficient recruitment and interaction with SHPRH, thus promoting mutagenic TLS at the expense of error-free TS. Surprisingly, USP7 also deubiquitylates PCNA (Kashiwaba et al., 2015). However, unlike the S phase-associated activity of USP1, USP7 activity toward PCNA was not found to be coupled to replication and was therefore proposed to prevent damage-induced mutagenesis during cell cycle-independent processes such as other DNA repair events. In summary, USP7 appears to be an important modulator of replication efficiency and fidelity not only during unperturbed replication, but also during DNA damage bypass.

### The Fanconi Anemia Pathway

DNA interstrand cross-links (ICLs) are strongly replication fork-stalling lesions that are not only refractory to copying by replicative DNA polymerases, but also prevent strand separation and passage of the helicase. Accordingly, their processing in replicating cells requires an intricate operation involving components of several repair pathways, namely TLS polymerases, HR proteins and structure-specific nucleases. In vertebrate cells, cooperation between these factors is mediated by the FA pathway, named after a rare hereditary disease associated with bone-marrow failure, congenital abnormalities, cancer predisposition and a marked sensitivity to ICL-causing agents (Kottemann and Smogorzewska, 2013; Walden and Deans, 2014). Nineteen genes have been assigned to this pathway by means of epistasis analysis, and eight of these encode subunits of a multimeric ubiquitin ligase, the FA core complex (Coleman and Huang, 2016). This E3 is recruited to chromatin upon stalling of the replisome upstream of an ICL, where its catalytic subunit, FANCL, monoubiquitylates a heterodimer of two other FA proteins, FANCD2 and FANCI (Alpi et al., 2008; Longerich et al., 2009; Sato et al., 2012). The ubiquitylated form of this “ID complex” initiates ICL processing, which involves the generation of a collapsed fork as a step toward the unhooking of the cross-link by dual incisions on either side of the lesion. It is followed by TLS-mediated repair synthesis and HR-mediated reactivation of the replication fork. How these downstream events are accomplished has only recently been elucidated. Central to the unhooking step is the scaffold protein, SLX4/FANCP, which recognizes the ubiquitylated ID complex by means of two ubiquitin-binding UBZ domains and interacts with a number of structure-specific nucleases that mediate the actual incisions (Zhang and Walter, 2014). Ubiquitin binding by SLX4 is required for cellular resistance specifically toward DNA cross-linking agents (Kim et al., 2013), and mutations in SLX4 confer a FA phenotype in humans, highlighting the importance of SLX4 for ICL repair (Kim et al., 2011; Stoepker et al., 2011).

Another structure-specific endonuclease, FANCD2/FANCI-associated nuclease 1 (FAN1), was identified to act downstream of the ID complex (Kratz et al., 2010; Liu T. et al., 2010; MacKay et al., 2010; Smogorzewska et al., 2010). Like SLX4, FAN1 carries a UBZ domain that was reported to mediate the recruitment to damage sites via binding to monoubiquitylated FANCD2. However, FAN1 was found to be dispensable for ICL incision in a cell-free system (Klein Douwel et al., 2014). Moreover, patients carrying a *FAN1* homozygous microdeletion do not suffer from typical FA conditions (Trujillo et al., 2012), thus arguing against a contribution of the nuclease to the FA pathway. Insight into this conundrum has very recently come from the observation that FAN1 instead prevents genomic instability induced by replication fork stalling events unrelated to ICLs (Lachaud et al., 2016). Thus, ubiquitylation of the ID complex by the FA core complex appears to serve a twofold purpose in response to replication stress, depending on the downstream effectors: a highly specialized ICL repair pathway triggered by SLX4 recruitment, and an independent, more general fork protection mechanism by means of FAN1.

Interestingly, the FA pathway appears to be intimately connected with another system for replication fork protection, the Rad18- and PCNA-dependent damage bypass mechanism described above. Not only does ICL processing require the activity of TLS polymerases, but the central initiating event of the FA pathway, the activation of the ID complex, was actually found to depend on Rad18, the E3 responsible for PCNA monoubiquitylation. The exact relationship between the two pathways is still a matter of controversy, as one study observed an interaction between FANCL and PCNA^Ub^ that was required for efficient recruitment of FANCL to chromatin (Geng et al., 2010), whereas another report postulated a direct role of Rad18 in binding and recruitment of FANCD2 in a manner independent of PCNA modification (Williams et al., 2011). Another piece of evidence for a tight coordination between the two pathways is the notion that the isopeptidase USP1 mediates deubiquitylation of both PCNA and the ID complex (Nijman et al., 2005; Huang et al., 2006). Controlling the ubiquitylation of these two key players appears to be essential for proper replication fork maintenance, as loss of USP1 causes high levels of genome instability and mutagenesis (Huang et al., 2006).

A recent study discovered a regulatory circuit of polyubiquitin and SUMO that also appears to contribute to controlling FA pathway activity at sites of replication problems (Gibbs-Seymour et al., 2015): upon treatment with replication fork-stalling agents, FANCD2 and FANCI are SUMOylated by two SUMO E3 ligases, PIAS1 and PIAS4, in a manner dependent on prior activation of the ID complex by monoubiquitylation. The modification targets the proteins for RNF4-mediated polyubiquitylation and subsequent extraction from the chromatin by the p97 segregase in complex with DVC1. Hence, this mechanism may limit ID complex dosage at the sites of replication stress in order to terminate the response or avoid excessive activity of the FA pathway.

## Replication of Chromatin

Genome replication occurs in the context of chromatin. Hence, for efficient copying of genomic DNA, nucleosomes must be disrupted ahead of an advancing replication fork. Upon passage of the fork, chromatin structure must rapidly be restored, and loss of epigenetic information in the process needs to be avoided. It is therefore not surprising that many chromatin components are targets of the ubiquitin and/or SUMO system for regulatory purposes, and these modifications are known to be important for the replication process itself.

### Ubiquitylation of Histones H2A and H2B

Histone H2A was the first protein discovered to be modified by ubiquitin (Goldknopf et al., 1975). In fact, H2A and H2B are two of the most abundant ubiquitylation targets in the nucleus (Cao and Yan, 2012). Both H2A and H2B are predominantly modified by monoubiquitin. H2B was found to be monoubiquitylated at K123 in *S. cerevisiae* or K123 and 120 in human cells, which plays an important role in transcriptional regulation (Henry et al., 2003; Wood et al., 2003; Kao et al., 2004; Nakanishi et al., 2009; Song and Ahn, 2010). In yeast, H2B monoubiquitylation is mediated by the E2 Rad6 and the E3 Bre1 (Robzyk et al., 2000; Wood et al., 2003). The mammalian homologs of Bre1, RNF20, and RNF40 (Kim et al., 2005), cooperate with the E2s hRad6 and UbcH6 (Koken et al., 1991). H2B^Ub^ promotes di- and tri-methylation of H3 at K4, which controls various aspects of transcription (Dover et al., 2002; Sun and Allis, 2002; Krogan et al., 2003), among them a stabilization of the histone chaperone complex FACT (Fleming et al., 2008).

**FIGURE 6:**
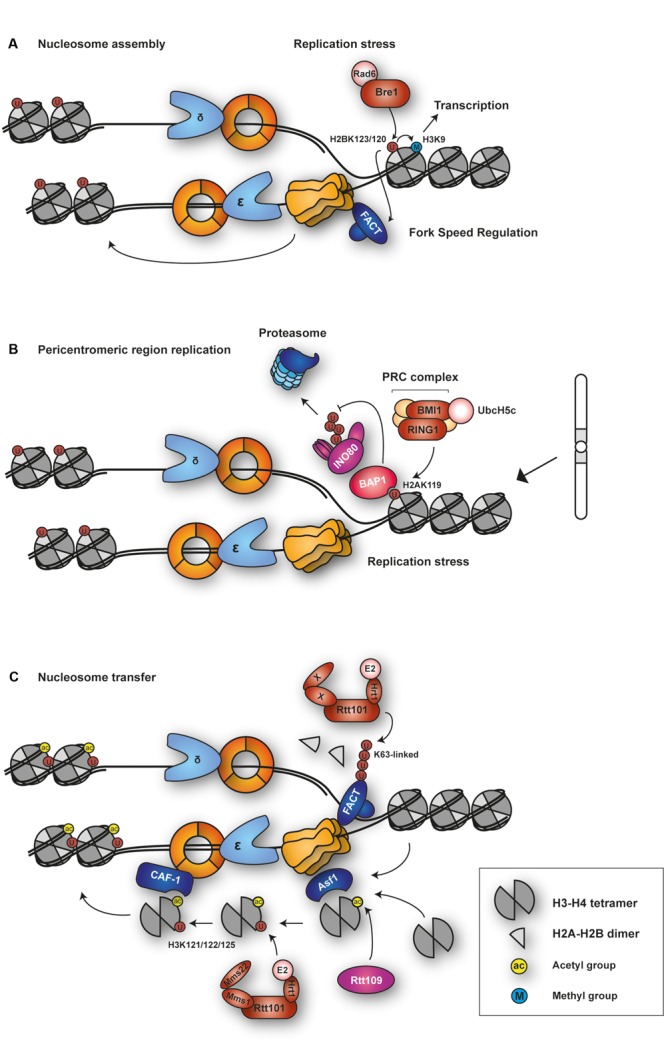
**Functions of histone ubiquitylation in DNA replication. (A)** Budding yeast E2–E3 complex Rad6-Bre1 is recruited to sites of replication stress for H2B ubiquitylation at K123. H2B^Ub^ regulates fork speed and nucleosome assembly behind the fork. Via H3K9 methylation, it independently contributes to transcriptional regulation. **(B)** The Polycomb Repressive Complex (PRC) is recruited to sites of replication stress or at problematic sequences to ubiquitylate H2A at K119. H2A^Ub^ recruits BAP1, which maintains fork stability by protecting chromatin-remodeling INO80 complex from proteasomal degradation. **(C)** The E3 SCF^Rtt101^ ubiquitylates H3 at K121, 122, and 125. The reaction is stimulated by acetylation of H3 at K56 by histone acetyltransferase Rtt109. This facilitates transfer of the H3-H4 tetramer to CAF-1 for nucleosome deposition behind the fork. Rtt101 also ubiquitylates the chromatin-reorganizing FACT complex.

Beyond its role in transcriptional regulation, H2B^Ub^ has been implicated in DNA replication (**Figure 6A**). This connection was established by the observation that Bre1 is enriched around replication origins, where it contributes to maintaining H2B^Ub^ levels on newly replicated DNA (Trujillo and Osley, 2012). Whereas a ubiquitylation-deficient mutant of H2B, K123R, is highly sensitive to replication fork-stalling agents (Trujillo and Osley, 2012; Lin et al., 2014), H3 mutants that abolish methylation are significantly less sensitive (Trujillo and Osley, 2012). This argues that the contribution of H2B^Ub^ to replication is independent of its regulatory role in transcription, mediated through histone methylation. In cells lacking H2B^Ub^, despite efficient formation of the pre-RC, association of replisome components such as polymerases 𝜀 and α and RPA with origins is impaired (Trujillo and Osley, 2012), as is replication progression after HU treatment (Lin et al., 2014). Also, PCNA associates normally at origins, but its levels are reduced at more distal sites, suggesting that the H2B-K123R mutant does not affect origin firing, but rather fork progression under conditions of replication stress (Trujillo and Osley, 2012). In addition, lack of H2B^Ub^ leads to a defect in the binding of the FACT complex and reduced nucleosome occupancy in newly replicated DNA under the same stress condition (Trujillo and Osley, 2012; Lin et al., 2014). H2B^Ub^’s effect on FACT in this context is reminiscent of its role during transcription. Since FACT is known to stimulate the activity of the Mcm helicase (Tan et al., 2006), it was speculated that H2B^Ub^ could play a role in facilitating the unwinding of DNA ahead of the fork to promote replication progression. However, this view was challenged by a recent report from Lin et al. (2014), who postulated that H2B^Ub^ may instead function to limit uncontrolled fork progression. They observed significant elongation of replication tracts in the absence of H2B^Ub^ after HU treatment, together with increased levels of H2A phosphorylation, a sign of fork damage (Lin et al., 2014). In support of this model, fork progression under conditions of replication stress is also strongly enhanced in *rad6*Δ cells (Yu et al., 2014).

Taken together, ubiquitylation of H2B appears to coordinate nucleosome assembly with fork progression, an activity that becomes particularly important when the replisome is challenged by replication stress such as nucleotide depletion or DNA damage. However, the precise mechanism and the effectors of the modification are yet to be defined.

H2A is well known to be ubiquitylated at the conserved residue K119 by the polycomb repressive complex 1 (PRC1), which comprises the RING-E3 subunits RING1A or RING1B and BMI1 together with the E2 UbcH5c (Gao et al., 2012; McGinty et al., 2014). The mark is essential for establishing repressive chromatin during development (Lanzuolo and Orlando, 2012; Di Croce and Helin, 2013). However, H2A^Ub^ may also play a role in the replication of intact and damaged DNA (**Figure 6B**). RING1B localizes to sites of replication (Lee et al., 2014; Piunti et al., 2014), and several Polycomb proteins were also found to be recruited to sites of DNA damage (Bergink et al., 2006; Chou et al., 2010; Ginjala et al., 2011), suggesting that H2A^Ub^ may contribute to damage signaling at replication forks. In fact, loss of PRC function causes an increase in asymmetric forks, indicating perturbed replication dynamics (Piunti et al., 2014; Bravo et al., 2015). Conversely, enhancement of H2A ubiquitylation by depletion of the ubiquitin-specific protease USP3 in mammalian cells causes delays in S phase progression and increased formation of ssDNA and DNA breaks (Nicassio et al., 2007). These observations are consistent with H2A^Ub^ acting as a damage signal that – when present in excess – leads to a hyperactivation of the damage response that would generate abnormal replication or repair structures causing genomic instability.

A recent study suggests a special role of H2A^Ub^ in the replication of pericentromeric heterochromatic domains, which are duplicated late in S phase (Bravo et al., 2015). Cells deficient in all RING1 activities were found to accumulate high levels of ssDNA in these regions, along with increased spontaneous levels of γH2AX and a delayed transition from middle to late S phase. Consistent with these findings, H2A^Ub^ colocalizes with PCNA in late S phase (Vassilev et al., 1995). Interestingly, selective restoration of H2A^Ub^ within the pericentromeric heterocromatic domains by means of a fusion construct of RING1B, BMI1 and methyl-CpG binding domain protein 1 (MBD1) rescued the defect in S phase progression in RING1-deficient cells (Bravo et al., 2015). Given the enrichment of major satellite repeats in pericentric heterochromatin and their propensity to form secondary structures, the strong effect of H2A^Ub^ in these regions may well reflect a general contribution of the modification to the replication of problematic sequences.

The mechanism by which H2A^Ub^ influences DNA replication is still unknown, but some insight comes from the observation that BRCA1-associated protein-1 (BAP1) recognizes H2A^Ub^ at replication forks and recruits the ATP-dependent chromatin remodeler Ino80. BAP1 deubiquitylates INO80 and thereby protects the protein from ubiquitin-mediated proteolysis (Lee et al., 2014). Hence, via BAP1 recruitment H2A^Ub^ might allow the INO80 complex to exert its well-known role in stabilizing stalled replication forks and assisting fork restart (Papamichos-Chronakis and Peterson, 2008; Shimada et al., 2008; Vincent et al., 2008; Falbo et al., 2009; Vassileva et al., 2014).

### Ubiquitylation of Histone H3 in Replication-Coupled Nucleosome Assembly

In order to ensure proper restoration of chromatin structure upon genome replication, the nucleosome assembly machinery is tightly coupled to replication fork progression. In budding yeast, this is achieved by means of a pathway involving acetylation of histone H3, a marker of newly synthesized histones (**Figure 6C**). In front of a replication fork, nucleosomes are disassembled by the action of the Mcm2-7 complex and histone chaperone Asf1 (Groth et al., 2007; Huang et al., 2015). Behind the fork, both parental and newly synthesized histones contribute to the restoration of chromatin structure. In *S. cerevisiae*, preferential binding of Asf1 to the H3-H4 dimer stimulates acetylation of newly synthesized H3 at K56 by the histone acetyltransferase Rtt109 (Masumoto et al., 2005; Driscoll et al., 2007; Han et al., 2007a). H3K56^ac^ enhances binding of H3-H4 to histone chaperones Rtt106 and CAF-1 (Li et al., 2008). CAF-1 in turn interacts with PCNA and assists in histone deposition behind the fork (Shibahara and Stillman, 1999; Moggs et al., 2000; Zhang et al., 2000). H3 acetylation peaks in S phase and is removed upon completion of genome replication (Masumoto et al., 2005). H3K56^ac^ is also detectable in mammalian cells, although in much lower abundance compared to yeast (Garcia et al., 2007; Das et al., 2009; Tjeertes et al., 2009; Yuan et al., 2009; Jasencakova et al., 2010), suggesting that either the modification is much more transient, or other acetylation sites can substitute for H3K56. Defects in the Asf1-Rtt109-H3K56^ac^ pathway result in various aspects of genome instability, including reduced replisome function under conditions of replication stress (Franco et al., 2005; Schulz and Tyler, 2006; Han et al., 2007b; Clemente-Ruiz et al., 2011), sensitivity to DNA-damaging agents (Driscoll et al., 2007; Li et al., 2008), loss of sister chromatid cohesion, excessive recombination and high rates of gross chromosomal rearrangements (Myung et al., 2003; Prado et al., 2004; Ramey et al., 2004; Thaminy et al., 2007; Kadyrova et al., 2013; Munoz-Galvan et al., 2013).

Intriguingly, a large-scale genetic screen in budding yeast identified the CRL ubiquitin ligase complex, Rtt101^Mms1/Mms22^, as a downstream effector of the pathway (Collins et al., 2007b). Rtt101^Mms1^ is believed to be the budding yeast ortholog of human CRL4^DDB1^ and assembles with the substrate adaptor Mms22 in a DNA damage-induced manner (Zaidi et al., 2008; Han et al., 2010, 2013). Inactivation of the complex causes damage sensitivity and defects in fork progression through damaged DNA and natural replication pause sites such as ribosomal DNA loci, and these defects are epistatic with the lack of Rtt109 (Luke et al., 2006; Duro et al., 2008; Zaidi et al., 2008; Wurtele et al., 2012). Moreover, Rtt109 was indeed found to recruit Rtt101 to chromatin (Roberts et al., 2008).

Despite the strong genetic link between the Asf1-Rtt109-H3K56^ac^ nucleosome assembly pathway and Rtt101^Mms1/Mms22^ in replisome functions and genome maintenance, their cooperation is not well understood in mechanistic terms. A recent report might provide insight into the process. Han et al. (2013) found that Rtt101^Mms1/Mms22^ preferentially binds H3K56^ac^-H4 over unmodified H3-H4 and can directly ubiquitylate H3 at lysine residues 121, 122, and 125. This in turn weakens H3 interaction with Asf1 and instead facilitates association with CAF-1 for subsequent deposition behind the replication fork. This function appears to be conserved in human cells, as depletion of CRL4^DDB1^ results in enhanced interaction of H3-H4 with Asf1 and reduced deposition of new H3 (Han et al., 2013). Hence, Rtt101^Mms1/Mms22^-mediated ubiquitylation of H3 appears to assist in a hand-off mechanism that ensures the transfer of H3–H4 from Asf1 ahead of an advancing fork to other chaperones such as CAF-1 and Rtt106 behind the fork.

How does nucleosome assembly influence replisome stability? There is growing evidence indicating that coupling of nucleosome assembly and replication progression is essential for maintenance of intact replisomes. This view is supported by the observation that deregulation of histone supply causes replication forks to collapse, followed by recombination-mediated rescue (Groth et al., 2007; Clemente-Ruiz and Prado, 2009; Takayama and Toda, 2010; Clemente-Ruiz et al., 2011; Mejlvang et al., 2014). This effect is reminiscent of the situation where lack of the Asf1-Rtt109-H3K56^ac^ or Rtt101^Mms1/Mms22^ pathway causes a decoupling of nucleosome assembly and replication progression.

Nevertheless, many mechanistic questions remain. For example, the majority of Cullin-based ubiquitin ligases are known to produce K48-linked polyubiquitin chains that target their substrates for proteasomal degradation (Komander and Rape, 2012; Mattiroli and Sixma, 2014), and the E2 Cdc34 that associates with Rtt101 is also known to assemble K48-chains (Ye and Rape, 2009). Yet, H3 itself is unlikely to be a substrate of the proteasome. This has led to the idea that Rtt101^Mms1/Mms22^ may target other components at stalled forks for degradation in order to facilitate repair or restart. Indeed, one such substrate could be the FACT complex (Han et al., 2010), which requires Rtt101 specifically for localization to sites of replication, but not to transcription sites. Intriguingly, however, in this case a K63-linked ubiquitin chain was detected on FACT (Han et al., 2010). Hence, it is still an open question whether Rtt101^Mms1/Mms22^ plays any role in proteasomal degradation mediated via K48-chains.

### Ubiquitylation of Histone H3 in Replication-Coupled Epigenetic Inheritance

In order to maintain its identity and gene expression patterns, it is crucial for a cell to restore its epigenetic information after every round of replication. Due to the semiconservative nature of DNA replication, DNA is hemi-methylated after every replication cycle, and full DNA methylation has to be restored in order to reestablish gene silencing. It is known that a RING-type ubiquitin ligase, UHRF1 (ubiquitin-like with PHD and ring finger domains 1, also known as NP95 in mouse and ICBP90 in humans), is recruited to nascent DNA after replication (Lopez-Contreras et al., 2013). UHRF1 binds to hemi-methylated DNA through its SET and RING finger-associated (SRA) domain (Arita et al., 2008; Avvakumov et al., 2008). Recently, Nishiyama et al. (2013) found that UHRF1 ubiquitylates histone H3 at K23 in *X. laevis* egg extracts. Methylation is then restored by DNMT1, which recognizes H3K23^Ub^ through its replication foci targeting sequence. A similar mechanism is observed in mammalian cells, where UHRF1 ubiquitylates H3 at K18. Here, DNMT1 binds to H3K18^Ub^ via a ubiquitin-interacting UIM motif (Qin et al., 2015). This is an interesting example of how cells can use the ubiquitin system to establish other epigenetic marks following DNA replication.

## Spatial Regulation of Ubiquitylation and Sumoylation During DNA Replication

**FIGURE 7:**
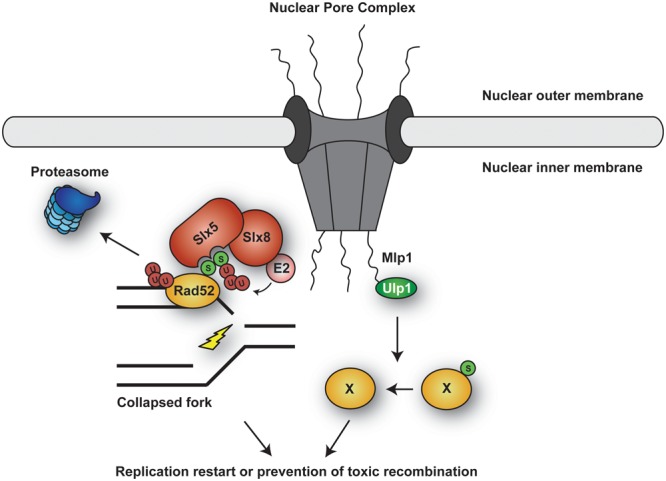
**Spatial organization of ubiquitin and SUMO metabolism in the nucleus.** Both ubiquitin and SUMO conjugating and deconjugating enzymes are enriched at the Nuclear Pore Complex (NPC). Collapsed forks relocalize to NPCs, which modulates local ubiquitylation and/or SUMOylation of relevant components in order to facilitate fork restart or prevent toxic recombination events.

The ubiquitin and SUMO systems are organized within the cell in a spatially controlled manner. One important hub for the coordination of nuclear ubiquitylation and SUMOylation activities appears to be the nuclear pore complex (NPC). The NPC is responsible for the transport of macromolecules between the nucleus and the cytoplasm, but genetic data from budding yeast suggest that it has additional functions in coordinating DNA damage signaling and repair (**Figure 7**). For example, it has been observed that cells deficient in components of the Nup84 nuclear pore subcomplex are hypersensitive to DNA-damaging agents (Bennett et al., 2001; Loeillet et al., 2005; Therizols et al., 2006) and accumulate spontaneous recombination foci in S and G2 phase (Loeillet et al., 2005; Palancade et al., 2007; Nagai et al., 2008). Mutations in both Nup84 and the HR pathway are synthetically lethal (Loeillet et al., 2005). These findings suggest that the NPC plays a role in replication during both unperturbed and stress conditions, and HR-based mechanisms to resolve fork problems become essential when NPC function is compromised.

A number of ubiquitin- and SUMO-related enzymes are found at the nuclear pore. For instance, SUMO protease Ulp1 is anchored to the nucleoporin Nup60 through myosin-like proteins (MLPs) Mlp1 and Mlp2 (Zhao et al., 2004). Mutation of *ULP1* or loss of MLPs shows synthetic effects when combined with mutations in HR (Zhao et al., 2004; Collins et al., 2007a; Palancade et al., 2007), and deleting MLPs leads to mislocalization of Ulp1, DNA damage sensitivity and clonal lethality (Zhao et al., 2004). Moreover, cells with impaired Ulp1function accumulate ssDNA during replication (Soustelle et al., 2004). It is therefore conceivable that the presence of deSUMOylating activity at the nuclear pore either prevents the accumulation of toxic recombination intermediates during replication or is required to resolve these structures. Proper localization of the SUMO conjugation system thus impinges on the process of DNA replication itself.

Intriguingly, the nuclear pore is also the site of accumulation of STUbLs in yeast (Nagai et al., 2011). Deletion of the STUbL complex Slx5/8 renders yeast hypersensitive to DNA-damaging agents and replication stress, and the mutants show higher rates of spontaneous gross chromosomal rearrangements (Zhang et al., 2006; Prudden et al., 2007; Nagai et al., 2008). Consistent with these findings, collapsed replication forks – like DSBs – are redirected to the nuclear pore (Nagai et al., 2008; Horigome et al., 2014). These observations prompted the hypothesis that relocalization to the nuclear pore facilitates HR-mediated fork restart by means of STUbL activity, possibly via degradation of SUMOylated proteins such as Srs2 (Urulangodi et al., 2015). In support of this idea, Su et al. (2015) recently observed that sites of replication blockage created by expanded CAG repeats are relocated to nuclear pores particularly in late S phase. The authors suggested that such relocalization may target Rad52^SUMO^ for degradation, which would then alter the outcome of HR pathways in the context of replication restart. In humans, it has been proposed that the PML nuclear bodies functionally resemble the yeast nuclear pores as a site where the mammalian STUbL RNF4 accumulates (Nagai et al., 2011). However, it remains to be tested whether perturbed replication forks are redirected to PML bodies in human cells.

Considering the large number of repair factors and replisome components that are SUMOylated during replication (Cremona et al., 2012; Psakhye and Jentsch, 2012), directing a collapsed fork to the nuclear pore may provide a window of opportunity for cells to fine-tune repair events by altering the fate of various repair and replication factors via posttranslational modification. However, it is still not fully understood how these activities are coordinated at the pore, for instance whether a certain factor is deSUMOylated by Ulp1 or directed to proteasomal degradation through STUbL activity. How such events would impact on the outcome of repair and the consequences for genome integrity awaits further investigation.

## Concluding Remarks

The extensive range of mechanisms by which ubiquitin and SUMO impinge on eukaryotic DNA replication is a very good reflection of the diversity of these two posttranslational modification systems in general. Several recurring concepts, including proteasomal targeting, either by ubiquitin alone or in a SUMO-dependent manner as mediated by the SUMO-targeted ubiquitin ligases, but also SUMO-mediated protection from ubiquitylation and proteolysis, can be observed to operate on replicating chromatin. Non-proteolytic functions, such as the enhancement of protein–protein interactions via SUMOylation, monoubiquitylation or linkage-specific polyubiquitylation, play an even more prominent role in the recruitment of various regulatory factors to active or stalled replisomes. Importantly, when compared to replication initiation, which is largely coupled to cell cycle regulatory events, replication fork progression appears to be an extremely delicate condition in which numerous modulating modifications are needed to fine-tune the activity of various components or stabilize weakly associated complexes in order to maintain fork integrity. No matter whether individual factors or entire groups of proteins are concerned, there is a large gray area between those modifications that regulate unperturbed replication and those that are initiated in response to replication problems and stress conditions.

Perhaps not surprisingly, a few key replication factors emerge as nodes in a network of posttranslational modification targets around the replication fork, such as PCNA, RPA and several central recombination proteins. Complementary to these prime targets, a few conjugation and deconjugation enzymes appear to dominate the replication-associated modification landscape and might thus be critical for coordinating different pathways involved in signaling or damage processing. These include ubiquitin ligases such as Rad18, RNF4 and the CRL4^Cdt2^ complex, but also prominent isopeptidases like USP1 and USP7. Undoubtedly, the range of identified targets and functions will continue to expand with the growing interest in these factors. Perhaps the biggest challenge for future research will be the interpretation of the wealth of information gathered by proteomics approaches. Substantiating and making sense of all the modification events that have by now been detected in system-wide screens, distinguishing relevant from bystander events, analyzing their regulation, and finally assigning a physiological role to them will occupy many laboratories for a long time to come.

## Author Contributions

All authors listed, have made substantial, direct and intellectual contribution to the work, and approved it for publication.

## Conflict of Interest Statement

The authors declare that the research was conducted in the absence of any commercial or financial relationships that could be construed as a potential conflict of interest.
